# Systematic review of structural and immunological features of mollusk toll-like receptors in aquaculture context

**DOI:** 10.3389/fimmu.2025.1671434

**Published:** 2025-10-01

**Authors:** Hongyu Li, Xianwei Li, Yunhuan Zhu, Jialu Xu, Qingzhi Zhao, Xintong Chen, Yijie Zhang, Ling Zhao, Yutong Chen, Ruiqi Weng, Keda Chen

**Affiliations:** Key Laboratory of Artificial Organs and Computational Medicine in Zhejiang Province, Shulan International Medical College, Zhejiang Shuren University, Hangzhou, China

**Keywords:** TLRs, mollusks, innate immune response, pattern recognition receptors, pathogens

## Abstract

**Introduction:**

Toll-like receptors (TLRs) are transmembrane proteins essential for innate immunity. While vertebrate TLRs have been well studied, knowledge of their distribution, structure, and function in mollusks remains limited, despite their importance in pathogen defense and environmental adaptation.

**Methods:**

This review synthesizes current findings on molluscan TLRs, focusing on their structural features, tissue-specific expression, immune functions, and responses to environmental pollutants.

**Results:**

Molluscan TLRs exhibit broad distribution and notable structural diversity. Their expression is tissue-dependent and can be modulated by pathogenic and environmental stressors. Although divergent from vertebrate TLRs in sequence homology, they share functional parallels in innate immune signaling. Evidence also indicates that pollutant exposure can alter TLR expression, influencing immune capacity.

**Discussion:**

Molluscan TLRs provide insight into species-specific immune strategies and hold potential for applications in disease management and ecological monitoring. Continued research will enhance understanding of innate immunity and support progress in comparative immunology.

## 1 Introduction

Traditionally, the immune system is divided into innate immunity and adaptive immunity, each with distinct functions and roles (1, 2). Innate immunity serves as the host’s first line of defense against microbial pathogen invasion. It is characterized by reliance on a limited set of receptors to detect invading pathogens, initiating rapid responses that typically trigger protective inflammatory reactions within minutes of pathogen exposure (3). Innate immunity is the sole defense mechanism by which invertebrates protect themselves against microbial pathogen invasion (4–6). Pattern recognition receptors (PRRs) constitute a crucial part of innate immunity, tasked with identifying a broad range of pathogen-associated molecular patterns (PAMPs) (7, 8). Certain PRRs bind directly to PAMPs, whereas others recognize molecules derived from PAMPs (7, 8). Mollusks possess diverse types of PRRs, such as C-type lectins and galectins, Gram-negative bacteria-binding proteins, scavenger receptors, peptidoglycan recognition proteins, and Toll-like receptors (TLRs) (9).

TLRs are widely distributed across many species and have been extensively studied in the innate immune systems of animals ranging from insects to humans (10, 11). The earliest identified TLR is Toll-1, which was recognized for its role in establishing dorsal-ventral polarity during embryonic development in *Drosophila melanogaster* (12–15). Currently, different numbers of TLRs have been identified in vertebrates. For example, 10 functional TLRs have been characterized in humans, 12 in mice, and 22 in chickens (16, 17). TLRs have a broad recognition spectrum and can detect diverse PAMPs derived from various microorganisms, including bacteria, viruses, protozoa, and fungi (1, 4). In vertebrates, upon recognizing PAMPs, TLRs activate intracellular signaling pathways that trigger innate immune responses, a critical step for initiating subsequent adaptive immunity (1). Mollusks rely almost entirely on innate immune mechanisms, which has led to remarkable diversity of TLRs within their immune systems. This diversity may be attributed to various evolutionary processes, including retrotransposition, gene duplication, high gene expansion rates, and alternative splicing of transcripts (4, 6). Since TLRs have been extensively studied in vertebrates, it is particularly important to investigate TLRs in mollusks, where research remains relatively limited.

Mollusks are classified into multiple classes based on their morphology and ecological habits, among which the most well-known are Gastropoda, Bivalvia, and Cephalopoda. Gastropods alone comprise over 100,000 extant species, accounting for approximately 80% of all mollusks (18). In the global marine aquaculture market, mollusk farming holds a significant share of total production, especially in the cultivation of edible shellfish (19). However, pathogen infections pose a significant threat to mollusk aquaculture (20). The primary causes of mortality include viruses, bacteria, and other microorganisms (20). Pathogens not only affect animal production but also pose potential risks to consumer health (21). In responding to these pathogens, studying TLRs in mollusks is particularly important. Since the vast majority of mollusks are marine filter feeders frequently exposed to various aquatic pathogens, their TLRs represent a meaningful subject for research (22).

This review aims to explore the diversity of TLR genes across different mollusk species and their critical roles in immune responses, further elucidating their potential applications in disease prevention and control within aquaculture. With ongoing advancements in understanding TLR downstream signaling pathways, research on TLRs holds promise for enhancing disease resistance and farming efficiency in cultured species. To our knowledge, this is the first systematic review applying the PRISMA framework to molluscan TLRs, highlighting their unique immunological features and aquaculture implications. Ultimately, these insights contribute to strengthening disease resilience in aquaculture systems and promoting sustainable industry development in harmony with broader socio-economic progress.

## 2 Method approach

A systematic review was conducted following the PRISMA 2020 guidelines. The objective was to synthesize recent findings on molluscan TLRs and the MyD88 signaling pathway and to assess their relevance to aquaculture. Literature published between January 2020 and May 2025 was retrieved from three databases: Web of Science, ScienceDirect, and PubMed. The following search terms and their plural forms were applied in various combinations: (“Toll-like receptor” OR “TLR”) AND (“mollusk” OR “mollusc”) AND (“functional analysis” OR “genome-wide” OR “whole-genome”) OR (“MyD88 pathway”).

After removing duplicates, records were screened based on predefined inclusion and exclusion criteria. Studies were included if they focused on mollusks, reported experimental data on TLRs or the MyD88 pathway, and were published in English in peer-reviewed journals. Exclusion criteria comprised duplicate publications, non-English articles, academic theses, technical or short reports, articles lacking original data, and studies with unavailable full texts. For each eligible article, information was extracted on publication year, mollusk species studied, novel TLRs identified or whole-genome analyses, downstream signaling pathways investigated, and key research findings (**Table 1**).

**Table 1:** Recently discovered novel TLRs and signaling pathways in mollusks, along with some whole-genome analyses.

Species	Year	Novel TLRs, genome-wide analyses of TLRs, and newly identified downstream signaling pathways	Discovery	Reference
*Chlamys farreri*	2024	*Cf*MyD88-2	Overexpression of *Cf*MyD88–2 in HEK293T cells activated IFN-α/β/γ and NF-κB reporter genes.	(23)
*Octopus sinensis*	2024	14 novel TLRs	TLRs were expressed at varying levels across different tissues, and most of them were upregulated in the examined tissues following stimulation.	(9)
*Zhikong scallops*	2024	*Cf*IKK2	The *Cf*IKK2 gene is expressed in multiple tissues and responds to stress induced by various pathogens. It interacts with the MyD88 protein, promotes homodimer formation, and its overexpression enhances MAPK phosphorylation and activates interferon reporter genes.	(24)
*Sinonovacula constricta*	2024	*Sc*TLR2	*Sc*TLR2 functions as a pattern recognition receptor capable of triggering immune responses to invading pathogens.	(25)
*Pinctada fucata*	2023	*Pf*TLR13	*Pf*TLR13 may play a critical role in inducing adaptive immunity and is associated with the acquired immune response in *P. fucata.*	(26)
*Zhikong scallops*	2023	*Cf*IKK3	The *Cf*IKK3 protein forms homodimers and interacts with CfIKK2, which may be a key step in activating itself and downstream transcription factors.	(27)
*Argopecten irradians* *Argopecten purpuratus*	2023	5 TRAF genes	*Ai*TRAF is abundantly expressed in the gills and hepatopancreas, suggesting it may play an important role in the scallop’s immune response.	(28)
*Haliotis discus hannai*	2023	29 TLRs	*H. d. hannai* possesses 143 single nucleotide polymorphism (SNP) sites with no insertion-deletion mutations (Indels). Eight TLR genes have been confirmed to be expressed in the hepatopancreas, gills, hemolymph, gonads, intestine, muscle, and mantle.	(29)
*Helix rufescens*		33 TLRs	There are 92 SNPs and 3 Indels, along with 6 missense mutations.	
*Homalogyridae laevigata*		16 TLRs	No SNPs or Indels were detected.	
*Crassostrea gigas*	2022	*Cg*MyD88s	*Cg*MyD88s, containing only the TIR domain, negatively regulates the NF-κB signaling pathway and plays an important role in antiviral immunity.	(30)
*Anodonta woodiana*	2022	*Aw*MyD88	*Aw*MyD88 is primarily localized in the cytoplasm of HEK293 cells and activates the NF-κB and AP-1 signaling pathways.	(31)
*Mytilus couscous*	2022	*Mc*TLR-like1 *Mc*MyD88a	It participates in the innate immune response to *Vibrio alginolyticus* and is involved in inflammatory responses via its interactions with TLR-like1 and MyD88.	(32)
*Hyriopsis cumingii*	2021	*Hc*TLRn	*Hc*TLRn is a member of the TLR family and may play an important role in activating the NF-κB signaling pathway in mollusks.	(33)
*Chlamys nobilis*	2021	4 novel *Cn*TRAF	*Cn*TRAFs are highly expressed in immune-related tissues and participate in the immune response following *Vibrio parahaemolyticus* infection.	(34)
*Ruditapes philippinarum*	2021	4 novel *Rp*TLR	*Rp*TLRs are primarily present in hemocytes and are significantly upregulated upon stimulation with *Vibrio anguillarum.*	(11)
*Radix Auricularia*	2021	10 novel TLRs	Identification of single- and multiple-cysteine cluster TLRs in species of the family Ampullariidae.	(35)
*Lymnaea stagnalis*		10 novel TLRs		
*Biomphalaria glabrata*		*Bg*TLR	These species possess Class 1-type TLRs, which were previously thought to be unique to *B. glabrata*.	
*Crassostrea gigas*	2021	*Cg*Toll-3	*Cg*Toll-3 may participate in the innate immune response against *V. alginolyticus* through a MyD88-dependent TLR signaling pathway.	(36)
*Pinctada fucata martensii*	2020	18 novel MyD88	The full-length sequence of *Pfm*MyD88–2 was obtained. *Pfm*MyD88–2 participates in the NF-κB signaling pathway and is regulated by *Pfm*miR-4047.	(37)
*Littorina littorea*	2020	45 novel TLRs	RNA-seq results from immune challenge experiments indicate that four TLRs and a set of signaling-related genes may be involved in the immune response of *L. littorea* against Digenean parasites.	(38)
*Anadara sativa*	2020	11 TLRs	Eleven TLR genes were identified and classified, and their expression levels in the hepatopancreas were found to be upregulated following lipopolysaccharides (LPS) stimulation.	(39)

**Figure 1:**
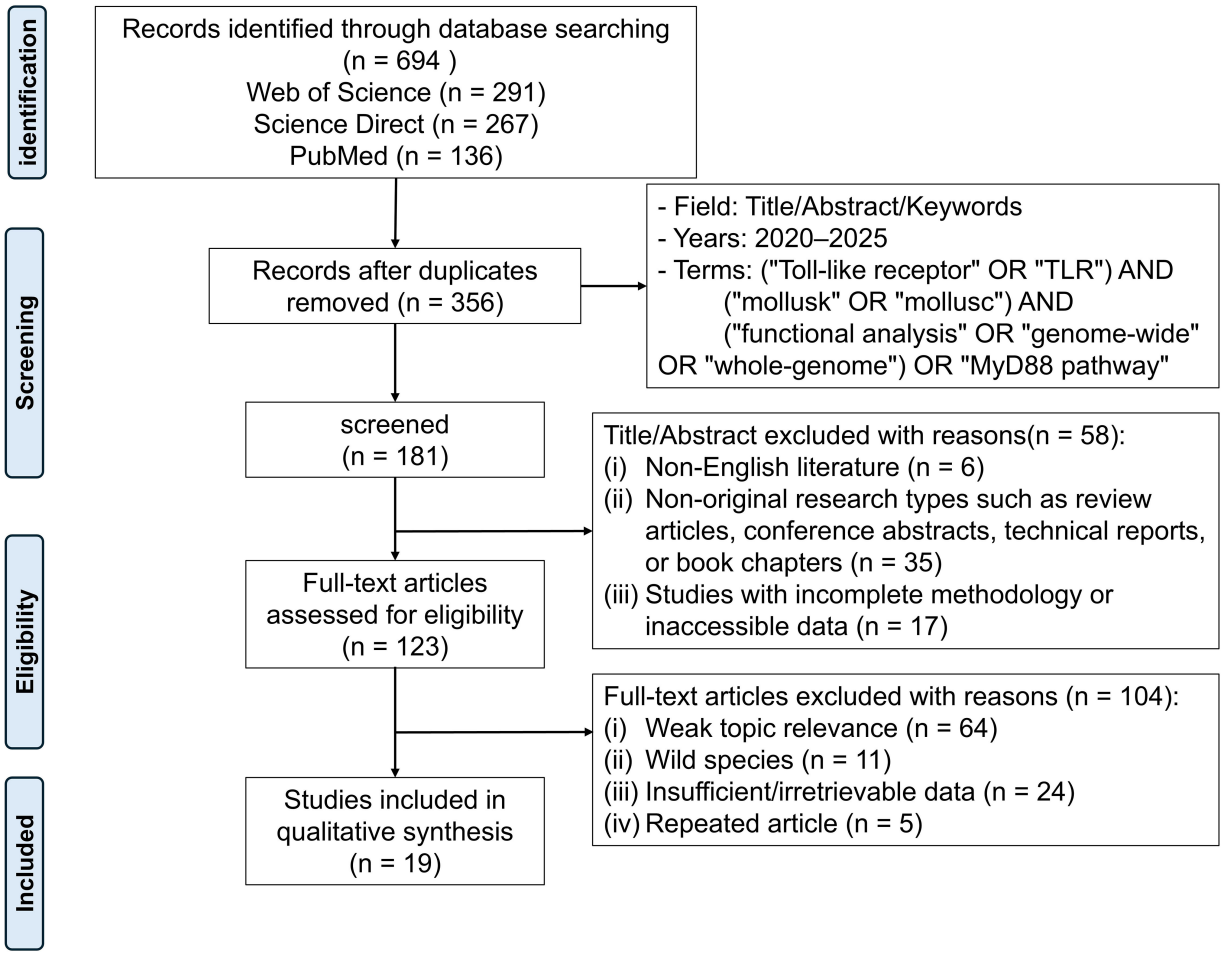
The PRISMA flow diagram illustrates the screening process of studies related to “TLR” and the “MyD88” pathway in mollusks, retrieved from the Web of Science, Science Direct, and PubMed databases between 2020 and May 2025. A total of 694 articles were initially identified. After removing duplicates, screening abstracts, and reviewing full texts, 19 eligible studies were finally included for analysis.

As the included studies were mostly descriptive and experimental, rather than randomized trials, no formal quality assessment was performed. The initial search yielded 694 records. After duplicate removal and application of exclusion criteria, 19 articles covering 24 mollusk species were retained for final analysis (**Figure 1**). The PRISMA flow diagram details the number of records excluded at each stage and the reasons for exclusion. This systematic review integrates these studies to provide a comprehensive overview of molluscan TLRs, their signaling pathways, and their functional implications for immune defense and aquaculture.

## 3 Structure and function of TLRs in mollusks

### 3.1 Unique structural differences of TLRs in mollusks

Upon recognition of external stimuli, TLRs initiate signaling cascades that activate downstream molecules, contributing to the innate immune response. Since innate immunity serves as the nearly sole immune mechanism in invertebrates, the robust immune defense systems of marine bivalves and other mollusks are crucial for adapting to the complex marine environment (40).

**Figure 2:**
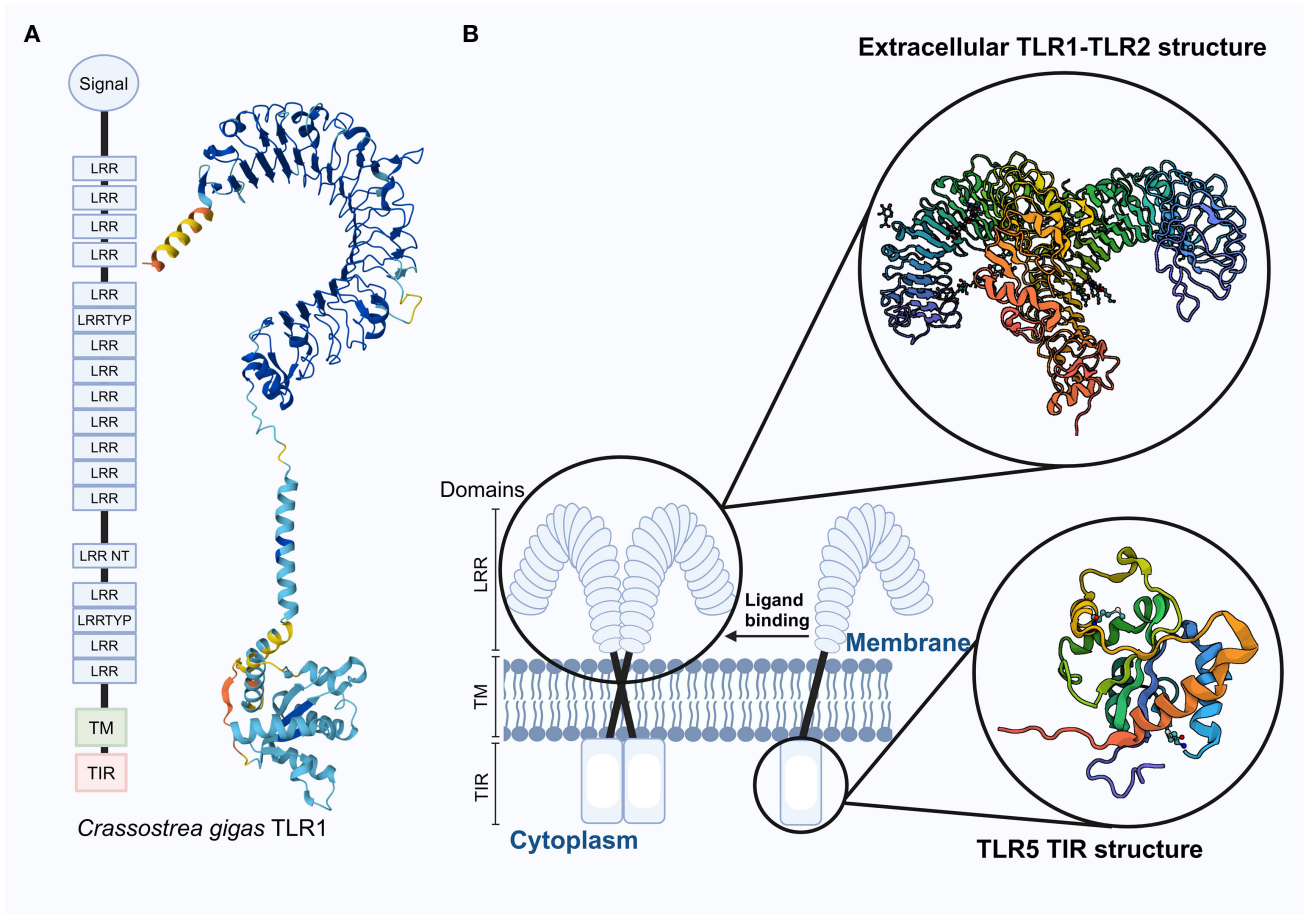
Distribution of the *Cg*TLR1 protein structure in the *Crassostrea gigas* with 3D prediction, along with a schematic diagram of the transmembrane structure of TLRs in vertebrates. **(A)** Functional domains of *CgTLR1* are adapted from (12), and the 3D structure on the right was predicted using AlphaFold2; it has not been experimentally validated in mollusks. **(B)** Schematic representation of vertebrate TLRs, showing the localization of TLR receptors within the cell membrane. The extracellular structure of the TLR1–TLR2 complex and the TIR domain of TLR5 are experimentally determined in humans (from NCBI). These vertebrate structures are shown for comparison only and should not be assumed to directly represent mollusk TLRs due to potential structural differences. Enlarged sections highlight the TLR1–TLR2 complex and the TIR domain of TLR5. Abbreviations: LRR, Leucine-rich repeat; LRRTYP, Leucine-rich repeat typical; LRR NT, Leucine-rich repeat N-terminal; TM, Transmembrane domain; TIR, Toll/Interleukin-1 receptor domain.

TLRs belong to the type I transmembrane protein family and consist of three main components: the extracellular region, which includes leucine-rich repeat (LRR) motifs forming a folded solenoid structure responsible for recognizing PAMPs; and the intracellular region, which contains a Toll/interleukin-1 receptor (TIR) domain that recruits various adaptor proteins to activate downstream signaling pathways (4). Known LRR family proteins contain multiple LRR motifs, ranging from as few as 2–3 to more than 40 (41). The dimerized LRR region forms an ‘m’-shaped structure, as shown in **Figure 2**, which is also compared with the TLR structures observed in vertebrates. The intracellular In addition to canonical TIR domains, researchers have identified a group of TIR-domain containing (TIR-DC) proteins in bivalve mollusks. These proteins exhibit high molecular diversity and may participate in multiple immune-related signaling pathways as well as embryonic development (42). These TIR-DC proteins exhibit significant molecular diversity among different species, and variable domain architectures and species-specific expansions are observed in different species (42). Phylogenetic analysis indicates that these proteins may have evolved through specific gene duplication and domain rearrangement events unique to mollusks (42). The experimental evidence regarding the functions of TIR-DC is still limited. Further research is needed, using methods such as RNA interference or CRISPR/Cas9 (once optimized in *Pyrrhocoris apterus*), to clarify their biological roles. The unique domain structure and expression profile of TIR-DC proteins highlight their potential as specific adaptors for the innate immune system in invertebrates, and provide a promising avenue for discovering new immune regulatory mechanisms.

Although research remains focused on a limited number of species, it is widely believed that TLR structures exhibit significant diversity in mollusks (22, 40). In invertebrates, the number and types of TLR family members vary across species, ranging from a single member to several hundred. To the best of our knowledge, several mollusk species have been identified their existing TLRs through whole-genome analyses, including but not limited to the following: *Strongylocentrotus purpuratus* has been reported to possess 222 TLR genes (43), *Haliotis discus hannai* contains 29 TLR genes (29), *Hippeutis rufescens* harbors 33 TLR genes (29), *Hippeutis laevigata* possesses encodes 16 (29), *Biomphalaria glabrata* possesses is known to have 56 TLR genes (44, 45), *C. gigas* 83 (44), and *Chlamys farreri* includes only 2 TLR genes (44). Additionally, we compiled newly identified TLRs and signaling pathways in mollusks from the past five years, as summarized in **Table 1**. The notable diversity exhibited by TLRs reflects the need for invertebrates to cope with increasingly complex and diverse environmental challenges and pathogenic pressures. Expansion of TLR families, particularly in bivalves, appears to result from multiple independent gene duplication events—especially tandem duplications—with frequent lineage-specific expansions occurring before and after bivalve radiation (22). These duplicated genes often experienced diversifying (i.e., positive) selection in their extracellular domains, enhancing variability in ligand‐binding regions and likely broadening the range of recognized pathogens (22). In addition, the broader evolution of TLRs across the Metazoa has been shaped by lineage‐specific expansions and losses, coupled with episodic positive selection in extracellular recognition domains—factors that collectively drive functional diversification in this receptor family (46). For instance, research on shallow-water mussels (*Mytilus galloprovincialis*) adapting to deep-sea conditions revealed differential expression of TLRs, suggesting that their expression and functional roles are modulated by varying environmental pressures (47). Similarly, in scallops (*C. farreri*), TLR genes are differentially upregulated upon bacterial and viral challenge via MyD88-dependent pathways, regulating antimicrobial peptide expression, and in abalone (*Haliotis cumingii*), HcToll1–3 are upregulated in response to pathogen stimulation, contributing to immune defense (48, 49). These examples collectively indicate that TLR diversity in mollusks reflects not only expansion in number but also diversification of function. Studies have shown that the TLR domains of green-lipped abalone and oysters share certain similarities, which may result from evolutionary divergence within the phylum Mollusca. Additionally, the presence of the TIR domain in both *Haliotis laevigata* and *C. gigas* indicates a degree of conserved homology among mollusk TLRs (50).

As a crucial component of the ancient immune system, TLRs are widely distributed across various organisms. The origin of TLRs predates the emergence of metazoans, and a single TLR gene encoding a receptor with a canonical structure exists in all metazoans (22). However, due to adaptive changes during evolution, we expect that mollusk TLRs exhibit certain differences in structure, function, and distribution compared to other species, and these differences may be associated to their ecological and biological characteristics. Compared to vertebrates, mollusk TLRs display greater multifunctionality and flexibility, which may stem from their extensive expansion and molecular diversification (6). This phenomenon may be related to gene duplication and domain rearrangement, among which tandem duplication frequently occurs in molecules associated to innate immunity (6, 31). Phylogenetic analyses indicate that the interactions between pathogens and the immune system have evolved over time, with selective pressures from specific pathogen recognition driving the optimization of TLR genes through both positive and negative selection (51). The early diversification of TLRs led to two major forms of the protein: in protostomes such as mollusks, TLRs typically contain multiple cysteine clusters, whereas in other deuterostomes, TLRs more commonly possess a single cysteine cluster (52, 53).

Studies ranging from invertebrates to mammals have shown that both TLR and NF-κB genes are widely present in the genomes of many animals, indicating that the NF-κB signaling pathway also has an ancient evolutionary origin and plays a vital role in the regulation of the immune system (52, 53). Sequence alignment based on amino acid sequences indicates that downstream genes of TLRs are more conserved across different species (12). This suggests that invertebrates may have undergone gene loss and diversification during the evolution of their immune systems.

### 3.2 Signaling pathway

**Figure 3:**
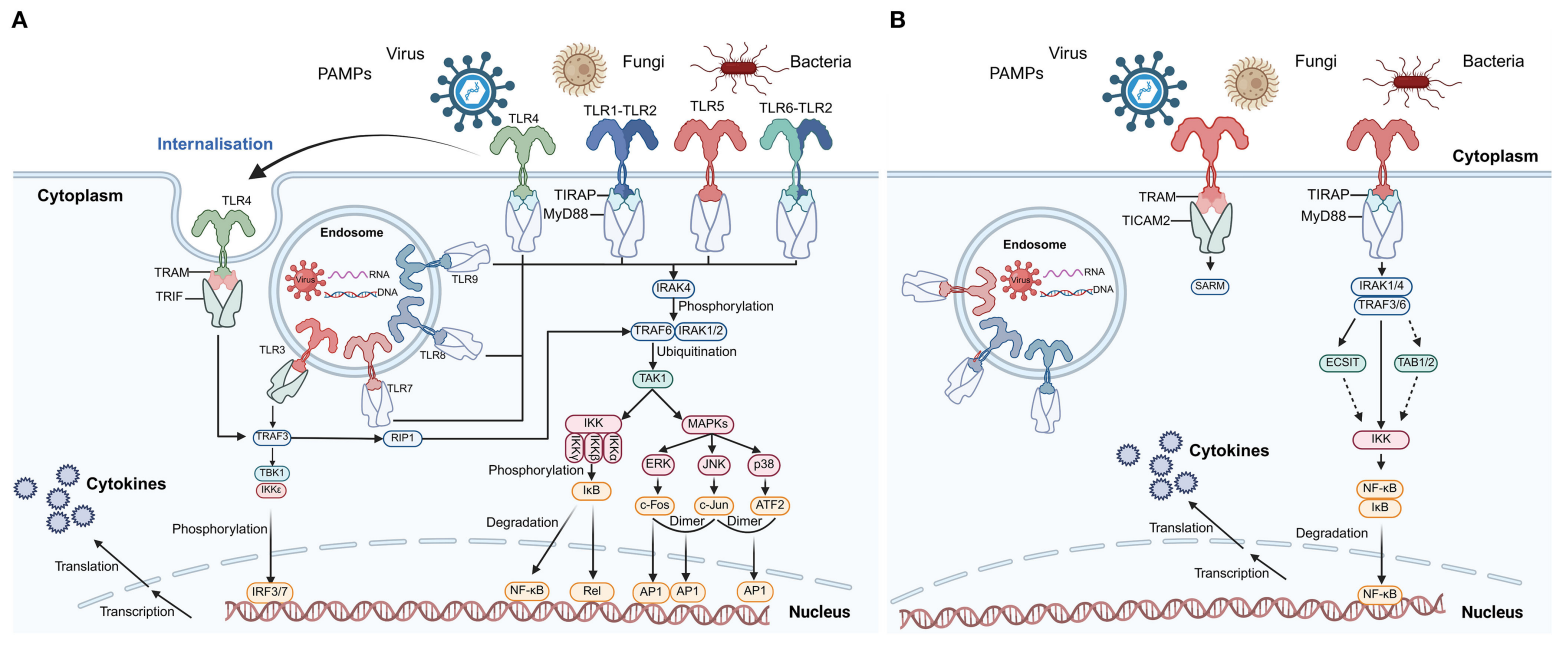
TLR signaling pathways in humans and *C*. *farreri*. **(A)** Human TLR signaling pathways. This panel illustrates the canonical TLR signaling cascades in humans, including both cell surface and endosomal receptors. Recognition of PAMPs from viruses, fungi, and bacteria activates TLRs, leading to recruitment of adaptor proteins such as MyD88, TRIF, TIRAP, and TRAM. Downstream signaling involves IRAKs, TRAFs, TAK1, the IKK complex, and MAPKs, culminating in the activation of transcription factors NF-κB, AP-1, and IRFs, which translocate to the nucleus to induce cytokine gene expression. Phosphorylation and ubiquitination events, as well as receptor internalization, are indicated. This panel is adapted from (55) and serves as a reference for vertebrate TLR signaling. **(B)** Putative TLR signaling pathways in *C*. *farreri*. This panel depicts the partially characterized TLR signaling in the mollusk *C*. *farreri*. Known components include TLR receptors, adaptor proteins (MyD88, TIRAP, TRAM, TICAM2), IRAKs, TRAFs, ECSIT, TAB1/2, and IKK, leading to NF-κB activation and cytokine production. Solid arrows represent interactions supported by experimental evidence, while dashed arrows indicate putative or indirect interactions. Pathway components were inferred based on two previous studies (10, 56). Abbreviations: PAMPs, pathogen-associated molecular patterns; NF-κB, nuclear factor kappa B; IRF, interferon regulatory factor; AP-1, activator protein 1; IKK, IκB kinase complex; MAPKs, mitogen-activated protein kinases; TRAF, TNF receptor-associated factor; IRAK, IL-1 receptor-associated kinase; TAK1, transforming growth factor-β-activated kinase 1; ECSIT, evolutionarily conserved signaling intermediate in Toll pathways; TAB, TAK1-binding protein.

TLRs are typical PRRs that recognize foreign pathogens, and their activation represents the first line of defense in the immune system. In vertebrates, TLR activation can recruit and activate neutrophils and macrophages (54). The TLR signaling pathway has been relatively well studied in humans, as shown in **Figure 3A**. The TLR family primarily functions through two signaling pathways: the MyD88-dependent pathway and the MyD88-independent pathway (also known as the TRIF-dependent pathway). In the MyD88-dependent pathway, MyD88 binds to TLRs through its TIR domain and recruits interleukin-1 receptor-associated kinase 4 (IRAK4). IRAK4 then phosphorylates and activates IRAK1/2 and TRAF6 (22, 57). Additionally, the MyD88 structure interacts with the TLR-TIR domain to activate the transcription factors AP-1 and NF-κB, thereby triggering inflammatory responses and initiating adaptive immunity (22, 57, 58). In the TRIF-dependent pathway, TRAM and other adaptor molecules interact with downstream components, including NF-κB, MAP kinases, and interferon regulatory factors (IRF3, IRF5, and IRF7) (59). Ultimately, the TLR signaling pathway triggers the expression of interferon-induced cytokines and various gene transcripts (59). However, due to the extensive diversity of TLRs in invertebrates, their mechanisms remain incompletely understood. The partially characterized TLR signaling pathways in mollusks are illustrated in **Figure 3B**. The TLR signaling network plays a critical role in immune defense by directly recognizing pathogens and PAMPs. Commonly used PAMPs include lipopolysaccharides (LPS), polyinosinic-polycytidylic acid [poly(I:C)], and peptidoglycan, which mimic bacterial and viral infections (60). For example, LPS stimulation has been shown to upregulate TLR transcripts in *C. gigas* and mussels *M. galloprovincialis*, suggesting a role in bacterial recognition and immune activation (61, 62). Similarly, poly(I:C), a synthetic analog of viral double-stranded RNA, significantly induces the expression of antiviral genes, including TLRs, in various mollusks (63). These PAMP-based stimulations are commonly used to dissect innate immune pathways and to investigate the functional responsiveness of molluscan TLRs. However, despite the increasing use of these stimuli, the downstream signaling mechanisms remain only partially elucidated, highlighting the need for further research on receptor-ligand specificity, signal transduction components, and cell-type-specific responses.

Recent studies have revealed a significant expansion of the MyD88 gene in mollusks, suggesting its potential functional diversity. For example, 19 MyD88-like genes have been identified in *Pomacea canaliculata*, 12 in *Haliotis rufescens*, 13 in *C. farreri*, 23 in *Mizuhopecten yessoensis*, 10 in *C. gigas*, and 7 in *B. glabrata* (6). In *C. gigas*, 27 proteins potentially interacting with TLRs have been identified, including three distinct MyD88 homologs, indicating that the TLR signaling pathway may involve a complex regulatory mechanism (64). In *C. farreri*, IKK proteins have been shown to play a key role in MyD88-mediated immune signaling, further supporting the functional conservation of the TLR pathway in mollusks as well as its potential lineage-specific regulation (65).

In mollusks, TLR-mediated inflammation plays a pivotal role in the innate immune response, particularly during the early recognition and elimination of pathogens. As a coordinated defense mechanism, this inflammatory response is essential for resisting infection (66). In recent years, studies on immune regulatory molecules in mollusks have been increasing. One such example is Octominin, a bioactive peptide derived from *Octopus minor*, which has been shown to effectively inhibit NF-κB transcriptional activation in LPS-stimulated RAW264.7 cells. It also dose-dependently reduces TLR4 mRNA expression, thereby suppressing TLR4-mediated inflammatory responses (66). This suggests that certain specific peptide molecules in mollusks can not only modulate TLR-mediated immune signaling, but also help maintain immune homeostasis through their immunosuppressive effects.

### 3.3 Expression of TLRs in mollusk tissues

Complete genome sequences have revealed a full set of PRRs in bivalves, which are generally associated to host defense against infections. For instance, in *Octopus sinensis*, TLRs are predominantly localized to the plasma membrane and show tissue-specific differential expression in hemolymph, white body, gills, and hepatopancreas, as revealed by RT-qPCR analysis (9). A separate study on *H. d. hannai* revealed that eight TLR genes are expressed across the hepatopancreas, gills, hemolymph, gonads, intestine, muscle, and mantle tissues. Notably, five TLRs were expressed in the gills, three in the hepatopancreas, and three in the hemolymph, with their expression levels significantly upregulated following *V. parahaemolyticus* infection (29).

In *C. farreri*, studies have shown that components of the TLR signaling pathway are constitutively expressed across various tissues, with particularly high expression in the gills and hepatopancreas (56). In the transcriptomic analysis of hemocytes and kidney tissues of *L. littorea*, 45 complete TLR transcripts were identified, which were derived from 35 distinct genes (38). Recent research on *P. fucata* revealed that the novel TLR13 (*Pf*TLR13) mRNA is ubiquitously expressed across all tested tissues, exhibiting the highest expression in the gills (26).

Furthermore, research has highlighted the involvement of TLR genes in mollusk development, which carries important significance for aquaculture practices. For instance, in *Haliotis diversicolor*, the MyD88 homolog gene (*hd*MyD88) exhibits ubiquitous expression throughout different embryonic stages (57). *Hd*MyD88 not only activates various downstream responses such as antioxidation, antibacterial activity, and apoptosis, but also plays a key role in immunity and development, which holds potential applications for improving survival rates and health status of abalone in aquaculture (57). Researchers have identified five TRAF genes in *Argopecten* and successfully cloned TRAF6. They analyzed the genetic features and phylogenetic relationships of these genes to enhance aquaculture management and disease control strategies in scallops (28). Initial research indicates that the mRNAs of Toll pathway signaling intermediates in *Crassostrea hongkongensis* exhibit broad expression in all tested tissues and throughout different embryonic developmental stages, potentially participating in embryogenesis and immune responses (67). Understanding the roles of these genes in oyster development and immunity can help optimize oyster aquaculture techniques, thereby improving farming efficiency and yield.

## 4 Pathogen infection and immune response in mollusks

### 4.1 Infection by pathogens and accumulation of microplastics

Mollusks have a certain capacity for bioaccumulation of pollutants such as viruses and bacteria within their bodies. Studies have shown that clams (*Ruditapes decussatus*), which inhabit shallow water environments and feed by filtering plankton and organic matter from the surrounding water, accumulate a large amount of pollutants in their bodies, including toxins, antibiotic residues, bacteria, viruses, and protozoa (68). Under high-density aquaculture conditions, the accumulation of these pollutants may lead to high mortality rates in mollusks (68). Another study specifically pointed out that the pathogenic microorganisms accumulated in clams mainly include *Vibrio cholerae*, *V. parahaemolyticus*, *Pseudomonas aeruginosa*, *Escherichia coli*, and *Enterococcus* spp (69). Direct experimental evidence demonstrates that exposure of *C. gigas to V. parahaemolyticus* rapidly up-regulates *Cg*TLR4 and activates the MyD88–NF-κB axis, leading to increased expression of antimicrobial peptides and pro-inflammatory cytokines (70). Similarly, functional studies using RNAi-mediated gene silencing have validated TLR-MyD88-dependent antibacterial responses in *C. hongkongensis* and *M. galloprovincialis* (71, 72).

Viruses typically cause widespread damage to mollusks, resulting in significant losses to the aquaculture industry. TLRs play a crucial role in combating viral infections by initiating antiviral immune responses that limit viral replication and spread. Viral infections in mollusks have been extensively studied, with the most common being Ostreid herpesvirus 1 (OsHV-1). In *C. gigas*, transcriptomic analyses revealed that TLR homologs, some lacking transmembrane domains, were significantly upregulated upon OsHV-1 infection, suggesting their potential involvement in antiviral responses (73). One study indicated that two genes encoding MyD88-like proteins, CgMyD88–1 and CgMyD88-2, as well as CgMyD88s, may all be involved in the TLR-mediated innate immune pathway (64). The study also found that CgMyD88s may act as a barrier to help prevent excessive inflammatory responses in *C. gigas* during infection with the OsHV-1 microvariant (64). Bivalves may bioaccumulate and be infected by human viruses within their tissues. Various viruses have been detected in bivalves, including members of the families *Papillomaviridae*, *Togaviridae*, *Picornaviridae*, *Bornaviridae*, and *Astroviridae* (74). These findings provide important guidance for disease prevention and management in the aquaculture industry. Protozoan pathogens can also activate TLR-mediated immune responses in mollusks, with *Perkinsus marinus* serving as a well-documented example. This parasite causes dermo disease in the *Crassostrea virginica*, leading to severe mortality in aquaculture. Comparative transcriptomic analyses between the resistant *C. gigas* and the susceptible *C. virginica* have revealed distinct host–parasite interactions across early and late stages of infection (75). Notably, *C. virginica* shows greater expansion and positive selection of TLR genes in response to P. marinus, reflecting lineage-specific diversification of pathogen recognition receptors (75). Furthermore, during early infection, oysters directly injected with *P. marinus* exhibited pronounced upregulation of innate immune transcripts, including TLR4 and TNF receptor-associated factor 6-like proteins, indicating that TLR-mediated signaling is rapidly mobilized under high parasite burden (76).

Furthermore, the impact of microplastics on mollusks should not be overlooked. Due to their persistence in the environment, microplastics can act as carriers for bacteria, facilitating the spread of potentially harmful pathogens to different habitats (77). Research has shown that exposure to microplastics alone can significantly inhibit the immune responses of blood cells in *Anadara granosa* and *M. galloprovincialis*, including reductions in hemoglobin concentration, total blood cell count, lysozyme activity, blood cell membrane stability, and the number of foot sacs (78, 79). At the same time, exposure alters reactive oxygen species (ROS) levels, calcium ion balance, and acid and alkaline phosphatase activities, exhibiting a clear dose-dependent pattern (78, 79). Microplastics can be internalized by cells, disrupting intracellular signaling pathways, disturbing immune homeostasis, and ultimately causing damage to tissues and organs. The generation of ROS represents a major mechanism of toxicity, which may further trigger the production of damage-associated molecular patterns (DAMPs) and is closely associated with the perturbation of TLR function, increased cytokine secretion, and inflammatory responses of immune cells (80). These findings highlight the potential threat of microplastics to mollusk health and the aquaculture industry, emphasizing the need for effective management and mitigation strategies.

### 4.2 Environmental stress and adaptation

TLRs ensure that cells effectively respond to environmental changes and pathogenic challenges. By initiating innate immune signaling cascades, TLRs ensure rapid and appropriate cellular responses under fluctuating environmental conditions. These receptors interact with a variety of regulatory molecules that fine-tune the magnitude and duration of signaling events, thereby maintaining immune homeostasis across diverse ecological contexts (81–83). In mollusks, the mechanisms of immune tolerance are believed to limit excessive inflammation and prevent self-damage, yet the role of TLRs in mediating such tolerance remains poorly understood due to a scarcity of systematic studies. Similar to vertebrates, molluscan TLRs are hypothesized to engage in crosstalk with other PRRs, orchestrating immune responses through synergistic activation, signal suppression, and dynamic feedback regulation, including both positive and negative loops (84, 85).

Mollusks are highly sensitive to endocrine-disrupting chemicals, which can disrupt immune homeostasis. MicroRNAs (miRNAs) play a central role in regulating TLR signaling and mediating immune responses to environmental and microbial challenges (86). For instance, in *M. coruscus*, LPS exposure downregulates miR-196, which normally targets *McTLR-like1* mRNA, resulting in increased *McTLR-like1* expression and a feedback mechanism that amplifies TLR signaling to enhance pathogen defense (87). Exposure to pollutants such as bisphenol A, 17α-ethinylestradiol, and tributyltin has been shown to alter immune-related miRNA profiles, disturb TLR pathways, and impair immune competence (88). Together, these findings highlight miRNA-TLR interactions as an adaptive mechanism enabling mollusks to cope with immunotoxic stressors in aquatic environments.

## 5 Disease control and ecological applications of molluscan TLRs

In the context of disease prevention and control, TLRs serve as key targets for enhancing immune responses and modulating immune memory, making them valuable for the prevention and treatment of diseases in aquaculture species. In Atlantic salmon and rainbow trout, for example, stimulation of TLR7/8 with synthetic agonists drives robust type-I IFN and pro-inflammatory cytokine production in the brain, kidney and spleen, providing a rational basis for TLR-centered vaccines (89). Beyond their canonical role in immune defense, TLRs also help mollusks cope with complex environmental stressors: activation of TLR-NF-κB pathways by thermal and hypoxic stress has been linked to improved survival under climate-change scenarios, while pollutant-induced TLR dysregulation serves as a sensitive biomarker for monitoring aquatic contaminants (90). Consequently, TLR-centered interventions are emerging as dual-purpose tools—both for aquaculture health management and for ecological risk assessment in rapidly changing environments.

### 5.1 Disease prevention and control

In oysters, specific TLRs have been identified that recognize PAMPs from viruses, subsequently triggering intracellular signaling cascades and inducing the production of antiviral molecules such as interferon-like proteins and antimicrobial peptides (91, 92). The use of TLR agonists to enhance immune responses in mollusks, as well as stimulating TLRs to improve vaccine efficacy, has become an important strategy for optimizing immune-based disease prevention and control (93). Nevertheless, their efficacy remains largely untested in mollusks. In vertebrates, TLR agonists such as monophosphoryl lipid A (TLR4 ligand) and CpG oligodeoxynucleotides (TLR9 ligand) have already been shown to enhance both mucosal and systemic immunity to vaccine antigens (94). Similarly, in vertebrates, synthetic TLR4 agonists can stimulate innate resistance to infectious challenges, while combinations of TLR2/6, TLR3, and TLR9 ligands have increased dendritic-cell activation and improved the protective efficacy of peptide vaccines in animal models (95). Although these mechanistic insights come largely from vertebrates, they provide a rationale for exploring TLR agonists as immunostimulants and vaccine adjuvants in molluscan aquaculture, with the understanding that their efficacy and safety in mollusks remain to be tested.

Based on studies of TLRs, vaccine adjuvants could potentially be used to enhance immune responses in mollusks and improve their immunogenicity. Protein-based adjuvants are capable of binding and activating PRRs, eliciting effective immune responses through the cooperation of innate and adaptive immune systems (96). Molluscan hemocyanins have been shown to interact with TLR4 in mammalian APCs, triggering MyD88-dependent signaling, cytokine release, and dendritic cell maturation (96). As adjuvant agonists, TLRs activating TLR signaling to promote antigen presentation, co-stimulatory signaling, and cytokine expression (97). Vaccine development in mollusks is still in its early stages, but its potential applications warrant further exploration and research. However, practical application in mollusks is currently limited by the lack of species-specific ligand detection methods, variable immune responses among mollusk species, and regulatory and cost considerations. In the future, research will need to optimize the assessment of the long-term efficacy and safety of the dosage.

### 5.2 Aquaculture and ecological applications

Given the important role of mollusks in the aquaculture sector, enhancing their immune capacity and reducing disease incidence have become key issues in both research and production. Although various aspects of TLRs have been described in some fish, shellfish, crustaceans (shrimps), and mollusks, their roles in other aquatic organisms remain largely unexplored (44, 98). Beyond their canonical role in immune defense, TLRs also help mollusks cope with complex environmental stressors. In the Pacific abalone *H. d. hannai*, transcriptomic profiling showed that both thermal and hypoxic stress rapidly up-regulate the TLR–NF-κB signalling cascade, and this activation was positively correlated with improved survival under simulated climate-change scenarios (99).

TLRs in mollusks can serve as valuable indicators for environmental and ecological monitoring. Exposure to bacterial or viral PAMPs rapidly alters TLR–MyD88–NF-κB transcript levels in hemocytes, changes that can be quantified by qPCR and exploited to optimize rearing conditions and reduce disease incidence in real time (100). Anchoring native TLR proteins on gold-nanoparticle or graphene-based electrochemical sensors has produced portable devices capable of detecting picogram-per-milliliter concentrations of LPS and viral dsRNA in seawater within minutes, providing farmers with precise, on-site data for timely management decisions (101).

Airborne pollutants may directly interact with receptors or indirectly modulate TLR signaling activation by generating secondary mediators such as DAMPs or PAMPs (102). Transplantation of TLR-hyper-responsive *Ruditapes philippinarum* into PAH-contaminated estuaries accelerated sediment microbial recovery and reduced pollutant loads by 28% within 90 days, demonstrating a practical mollusk-based strategy for ecological restoration (103). Nevertheless, the widespread application of TLR-based monitoring and interventions in aquaculture still faces challenges including species-specific variability, the need for standardized assays, cost, and regulatory constraints. Analyzing molluscan TLR expression in response to environmental pollutants may inform the development of immune-based strategies for ecological restoration, enhancing ecosystem resilience.

## 6 Conclusions and perspectives

Aquatic environments are important sources of pathogenic microorganisms, providing favorable conditions for the growth of bacteria, fungi, viruses, and other aquatic microbes, which can lead to mortality in aquatic animals. Mollusks, as representative aquatic organisms, ingest these pathogens and form part of the food chain, thereby posing certain risks to global economic development and human health. At present, due to the diversity of the TLR gene family, significant interspecies differences exist, and the expression and function of TLRs in mollusks are influenced by various factors. Therefore, further research is needed to broaden our understanding of TLRs in mollusks. Molluscan TLRs may also have the potential to be selectively activated by specific ligands or agonists. Similar to vertebrates, these ligands or agonists can bind to and activate TLRs, thereby enhancing immune responses in a manner comparable to vaccine adjuvants. However, this possibility still requires validation in mollusks. Research priorities should focus on addressing major knowledge gaps in molluscan TLR studies, such as the lack of functional validation for many identified TLR genes, interspecies differences, and the establishment of ligand screening and identification methods. Advancing studies on molluscan TLRs will be of significant value in enhancing our understanding of species-specific immune mechanisms and promoting progress in comparative immunology.

## Data Availability

The original contributions presented in the study are included in the article/supplementary material. Further inquiries can be directed to the corresponding authors.
